# Assessing the Privacy of mHealth Apps for Self-Tracking: Heuristic Evaluation Approach

**DOI:** 10.2196/mhealth.9217

**Published:** 2018-10-22

**Authors:** Luke Hutton, Blaine A Price, Ryan Kelly, Ciaran McCormick, Arosha K Bandara, Tally Hatzakis, Maureen Meadows, Bashar Nuseibeh

**Affiliations:** 1 Software Engineering and Design Group School of Computing and Communications The Open University Milton Keynes United Kingdom; 2 Microsoft Research Centre for Social Natural User Interfaces University of Melbourne Melbourne Australia; 3 Trilateral Research Ltd London United Kingdom; 4 Faculty Research Centre for Business in Society School of Business Coventry University Coventry United Kingdom; 5 Lero University of Limerick Limerick Ireland

**Keywords:** privacy, usable security and privacy, mHealth apps, mobile phone

## Abstract

**Background:**

The recent proliferation of self-tracking technologies has allowed individuals to generate significant quantities of data about their lifestyle. These data can be used to support health interventions and monitor outcomes. However, these data are often stored and processed by vendors who have commercial motivations, and thus, they may not be treated with the sensitivity with which other medical data are treated. As sensors and apps that enable self-tracking continue to become more sophisticated, the privacy implications become more severe in turn. However, methods for systematically identifying privacy issues in such apps are currently lacking.

**Objective:**

The objective of our study was to understand how current mass-market apps perform with respect to privacy. We did this by introducing a set of heuristics for evaluating privacy characteristics of self-tracking services.

**Methods:**

Using our heuristics, we conducted an analysis of 64 popular self-tracking services to determine the extent to which the services satisfy various dimensions of privacy. We then used descriptive statistics and statistical models to explore whether any particular categories of an app perform better than others in terms of privacy.

**Results:**

We found that the majority of services examined failed to provide users with full access to their own data, did not acquire sufficient consent for the use of the data, or inadequately extended controls over disclosures to third parties. Furthermore, the type of app, in terms of the category of data collected, was not a useful predictor of its privacy. However, we found that apps that collected health-related data (eg, exercise and weight) performed worse for privacy than those designed for other types of self-tracking.

**Conclusions:**

Our study draws attention to the poor performance of current self-tracking technologies in terms of privacy, motivating the need for standards that can ensure that future self-tracking apps are stronger with respect to upholding users’ privacy. Our heuristic evaluation method supports the retrospective evaluation of privacy in self-tracking apps and can be used as a prescriptive framework to achieve privacy-by-design in future apps.

## Introduction

The quantified self (QS) movement refers to the use of self-tracking technologies to capture data about different facets of a person’s life [1]. Recent advances in sensing technologies have allowed people to track a variety of life attributes, ranging from physical exercise to mood, sleep, and work productivity [2]. Collecting these data allows people to engage in self-improvement or behavioral change or to satisfy intellectual curiosity [3]. In the medical domain, self-tracking is increasingly being used to support health-related outcomes, with patients using data to reflect on their recovery [4] and clinicians deploying tracking technologies to monitor patients [5].

While many of the services that enable self-tracking are free or low cost, users may unwittingly be paying a price by surrendering their privacy to these services [6]. That is, users of self-tracking apps may find that their privacy is eroded because they lack control over how their data are collected, stored, and analyzed by self-tracking apps, and they may, in turn, have no say in how these data are shared with third parties. These issues point toward a need to understand privacy issues in self-tracking apps, particularly mobile health (mHealth) apps, which collect medical and health-related data. Such technologies frequently operate in an uncertain regulatory space and may not be afforded the protections and scrutiny that are given to other medical data, for example, the Health Insurance Portability and Accountability Act in the United States or National Health Service oversight in the United Kingdom.

Research has shown that self-tracking apps can give rise to privacy concerns. For example, apps that collect data about dementia often fail to disclose how the data are processed [7], and the vast majority of mHealth apps have, at least, some potential for information security and privacy infringements [8]. Other work has examined the privacy policies of self-tracking app vendors and identified privacy concerns [9]. However, these studies only focused on a small subset of apps. Recent years have witnessed increased public awareness of privacy issues [10-12], and users are known to be concerned about the improper use of sensitive data [13]. This suggests a need to evaluate the broader landscape in order to provide a characterization of privacy risks that can emerge in mHealth apps that are intended for self-tracking. Understanding potential privacy risks will, then, allow designers to account for such issues when creating self-tracking apps in the future.

However, currently, there is a lack of techniques for evaluating the privacy-related features of self-tracking apps. While researchers have developed frameworks for eliciting privacy requirements and achieving privacy-by-design [14-16], these are general frameworks that do not consider the specifics of self-tracking and mHealth apps. Similarly, privacy impact assessments [17-19] can offer a generalized analysis for large systems but are not designed for issues specific to mHealth apps. The Data Protection Impact Assessments mandated by Article 35 of the European Union’s (EU’s) General Data Protection Regulation (GDPR) [20] specify that the assessment must be systematic but give no guidance regarding the method or, even, regarding whether health-related data, such as those in mHealth apps, need special treatment. Recent privacy frameworks that focus on the Internet of Things [21] are closer to the mHealth domain, but these frameworks provide few insights into the effectiveness of features that are intended to control the sharing and access of personal data in self-tracking apps.

The aim of this study was to understand how current self-tracking apps perform with respect to privacy. To do this, we used a set of 26 heuristics to evaluate privacy in self-tracking technologies. These heuristics span 4 dimensions: notice and awareness, choice and consent, access and participation, and social disclosure usability. The heuristics are intended to support systematic appraisal of the ways in which self-tracking apps can either uphold or impinge on users’ privacy. We demonstrated the practical value of the heuristics by evaluating the privacy-related features of 64 popular self-tracking apps. Furthermore, we identified which dimensions of privacy were best met and explored whether certain types of services performed better than others. In addition, we examined a number of nonhealth-related apps, which enable self-tracking, allowing us to determine whether mHealth apps exhibit distinctive privacy characteristics.

The paper makes two contributions. First, the heuristics provide a low-cost approach for evaluating privacy in mHealth services, building on the use of the heuristics in studies of privacy in other domains [22-25] and extending the work of Furano et al [26], who developed a set of privacy heuristics for evaluating personal health records. Our heuristics can support the evaluation of self-tracking apps more generally and will allow researchers to gauge the ways in which privacy is met in future apps. Furthermore, the heuristics can provide a prescriptive privacy framework for designers, allowing them to achieve privacy-by-design in new mHealth services.

Our second contribution is an investigation of the state of privacy in mHealth services. We found that the category of data collected by an app is not a useful predictor of its overall privacy score, suggesting that poor support for privacy is an issue that pervades the self-tracking landscape. In addition, we showed that mHealth apps perform worse than other types of self-tracking apps in terms of privacy. This draws attention to the need for careful scrutiny of privacy practices within these apps, as well as the need to develop standards to ensure that mHealth apps pay sufficient regard to user privacy in the future.

## Methods

### Developing the Heuristics

Our heuristic analysis focuses on the information and controls given to the users to help them decide whether they wish to use a particular app, and which later help them selectively disclose their data to others. This includes the app’s user interface as well as its terms of service and privacy policies. Our heuristics do not focus on the “invisible” facets of privacy, which can affect end users, such as the sharing or selling of data without users’ consent; this is because it is difficult to assess whether these outcomes will arise when deciding to use an app. We, therefore, focused on what is known about an app at the point of use, given the claims made in its supporting documentation and the user interface design.

Our heuristic analysis addresses four key questions that users may ask when using self-tracking apps:

1. Am I informed about what happens to my data before I use this app?
2. Do I have control over my data once I start using the app?
3. Do I have access to the data I have provided?
4. Can I use features that allow me to control the disclosure of data to third parties?

**Figure 1:**
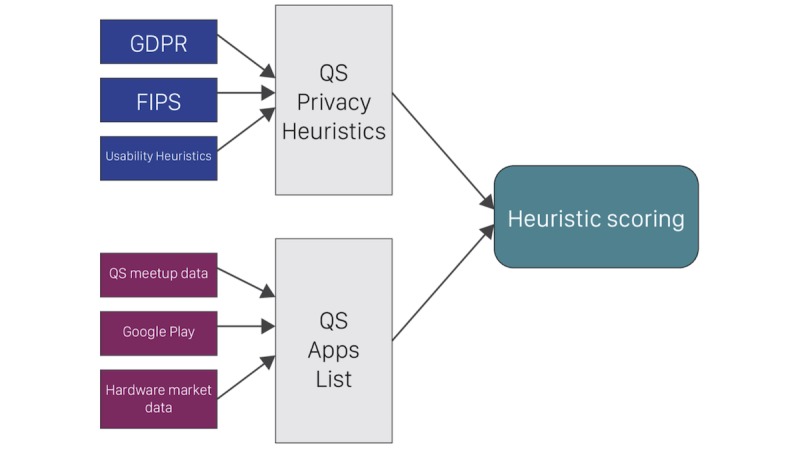
How the regulatory and market landscape was fed into the design of the heuristics and the choice of apps to study. GDPR: General Data Protection Regulation; FIPS: Fair Information Practices; QS: quantified self.

With these questions in mind, we began by reviewing sources in the regulatory landscape and relevant privacy literature (Figure 1; Multimedia Appendix 1) [27]. First, we used 3 categories from the FTC’s Fair Information Practices [28] policy as an initial guiding framework; these were *notice or awareness*, *choice or consent,* and *access or participation.* Each of these addresses a different aspect of information privacy (Table 1).

To populate these categories with appropriate heuristics, we considered some of the primary privacy concerns that manifest in self-tracking, such as ambiguous ownership and access to data, the significance of where data are stored, whether consent to store and process data can be revoked, and changes in privacy policies [29]. We then considered the EU’s GDPR, which updates and harmonizes data protection legislation across the EU, and incorporated considerations from this into our framework. Example provisions include the requirement for data to be portable, unambiguous consent, and the right to be forgotten [30]. In addition, we drew on the STRAP Framework [22], a technique that is aimed at supporting analysts in identifying privacy and security concerns during early design.

Finally, we used Inostroza et al’s usability heuristics for touchscreen mobile devices [31] to define the category of *social disclosure usability,* which refers to the usability of features that allow users to share QS data with a third-party service, for example, a social networking site (SNS). These heuristics assess concerns including the ability to prevent errors and the availability of support, both of which are relevant when considering interfaces that permit the disclosure of QS data. For example, an interface for supporting disclosure may include features for selecting the audience and subsets of content to share. Difficulty in the use of these features may lead to errors in sharing, causing privacy violations.

An initial set of heuristics was pilot-tested by 3 reviewers, all of whom were experts in HCI or software engineering and had personal experience of using self-tracking apps. The pilot tests explored whether the heuristics could be applied consistently across different apps and whether they were sufficiently exhaustive to capture the interactions encountered. Examples of improvements that arose from these tests included the rearrangement of 4 heuristics to better fit the Fair Information Practices categories and the addition of 1 heuristic to assess the granularity of disclosures to social networking services.

These improvements led to a final set of 26 heuristics (Multimedia Appendix 1) [27]. The heuristics include a combination of dichotomous and ordinal items, where a higher score for each item indicates better privacy-preserving characteristics within the target app. Figure 1 shows how the regulatory and market landscape led to the choices of heuristics and apps for this study.

**Table 1:** The 26 heuristics (H) present in our evaluation technique.

Category description and number^a^		Heuristic
**Notice or Awareness: These heuristics concern what people are told about how their data are used before their personal information is collected, such as the terms of service or privacy policies.**		
	H1	Before data are shared with a remote actor, the entity collecting the data is explicitly identified.
	H2	Before data are shared with a remote actor, the uses of the data are explicitly identified.
	H3	Before data are shared with a remote actor, the potential recipients are explicitly identified.
	H4	The nature and means of the data collected are explicitly identified.
	H5	Steps taken to ensure confidentiality, integrity, and quality of data are explained.
	H6	For those of above satisfied, notice is sufficiently explicit.
	H7	Can control when data are used for nonoperational secondary use, such as marketing or research.
**Choice or Consent: These include the controls people have over use of their data, such as whether to permit secondary uses of their data, including marketing.**		
	H8	Consent acquired before data shared with remote actor.
	H9	Consent is explicitly opt-in: no preticked checkboxes, etc.
	H10	Can choose which data types are automatically collected from sensors or other sources, for example, connect a finance app to a single bank account or track steps but not heart rate.
	H11	Data collection consent is dynamic: if new types of data are being collected, consent is renewed *in situ*.
	H12	Data processing consent is dynamic: if the purpose of processing changes, consent is renewed.
	H13	Data distribution consent is dynamic: if the actors’ data are distributed to changes, consent is renewed.
	H14	Consent to store and process data can be revoked at any time: with the service and any other actors.
	H15	Can control where data are stored.
**Access or Participation: These address issues such as whether people are able to view the data they have provided and whether they can verify its accuracy in a timely manner.**		
	H16	All raw collected data can be extracted from the service (in-app or via vendor’s website).
	H17	All data are available in standard text formats (CSV^b^, XML, JSON^c^, GPX^d^, etc).
	H18	Data extraction is available from within the service, for example, without raising a request with support.
	H19	Programmatic access to data is possible, for example, app programming interfaces are exposed.
**Social Disclosure Usability: These relate to the usability of interface elements that allow users to share data with third-party services, for example, social networking sites.**		
	H20	Privacy controls are per-disclosure, for example, individual workouts can be published to a social networking site, not relying solely on global defaults.
	H21	Privacy controls allow granular sharing of data types, for example, when sharing a workout, the distance can be shared but not the pace.
	H22	Error prevention: is explicit confirmation acquired before a disclosure?
	H23	Minimize user memory load: Effects of a disclosure are visible throughout the disclosure flow (ie, memory of earlier decisions not required).
	H24	Minimalist: During the disclosure flow no extraneous information (such as adverts or irrelevant user interface elements) is displayed.
	H25	Consistency: Information shown during the disclosure flow is consistent with the effect of the disclosure.
	H26	Help and documentation: Contextual help with making privacy decisions is available.

^a^See Multimedia Appendix 1 for the scoring criteria.

^b^CSV: comma-separated values.

^c^JSON: JavaScript Object Notation.

^d^GPX: GPS eXchange Format.

**Table 2:** The categories of apps used in our evaluation and the constituent search keywords for each group.

Purpose and app category		Keywords
**mHealth**		
	Cycling	Cycling
	Diet	Diet, eating
	Exercise	Exercise, workouts
	Apps with wearable hardware	N/A^a^
	Heart	Heartrate, heart rate
	Mood	Mood, happiness
	Running	Walking, running
	Sleep	Sleep
	Step count	Steps
	Weight	Weight, body fat
**Other self-tracking metrics**		
	Spending	Spending, income
	Time	Time keeping

^a^N/A: not applicable.

### Using the Heuristics to Evaluate Self-tracking Apps

To demonstrate the value of the heuristics for assessing privacy, we conducted a formal evaluation using a subset of mHealth and self-tracking apps from the Google Play Store. We focused on this platform because the Android mobile operating system was installed on approximately 85% of new smartphones shipped worldwide from 2016 to 2017 [32] and because Google’s policy for vetting apps is less restrictive than Apple’s or Microsoft’s policy [33], making it an attractive platform to collect a broad range of self-tracking apps.

To gather apps for our analysis, we used the responses to a 2014 survey of 105 members of the London Quantified Self Meetup Group [34] (Multimedia Appendix 2) to identify the types of data they collected. We then translated these into potential categories of apps to inform our search. We did this because no comprehensive taxonomy of self-tracking apps currently exists and because the London Group is one of the most well-established QS groups with a large number of early adopters who have experience with a broad range of tracking technologies.

After aggregating and ranking the survey responses, we used the 20 most-frequently reported types of data to produce a series of keywords such as “weight,” “sleep,” and “mood.” These keywords were supplied as input to a script that performed a keyword search on the Google Play Store. Each keyword search returned a list of apps relevant to that term. Next, metadata about each app was parsed from the Google Play Store and saved to a CSV file.

Our search produced an initial list of 345 apps; this was reduced to 292 after excluding instructional apps that demonstrate how to perform an exercise correctly. We excluded these apps because they do not capture data that can permit self-tracking. After this, we used market data from International Data Corporation via Statista [35] to identify major commercial vendors whose apps were not included in our search. Identified vendors were Xiaomi and Jawbone, whose Mi Fit and UP apps were manually added to the dataset. (The code used to produce this dataset and the dataset used in this analysis are available in a public repository [36].)

To sort the 292 apps into categories, we grouped keywords on the basis of similarity and characterized each app as either pertaining to mHealth or other self-tracking activities. Table 2 shows the outcomes of this classification and lists the 12 categories with the associated keywords that were used in this study.

To narrow the scope of our privacy evaluation and make the process more manageable, we elected to focus on a subset of the 292 apps. We did this by selecting the 7 most popular apps in each of the 12 categories (or as many as possible in categories with <7 apps); this resulted in a final list of 64 apps [37] (Multimedia Appendix 3).

Each of these apps was allocated to 1 of 4 reviewers to apply the heuristics independently. These reviewers had experience with self-tracking and evaluation of software systems, meaning that they were appropriately skilled to perform the evaluation. Apps were installed on an Android smartphone running Android 6.0 (chosen because it was the most popular version throughout 2017 [38]). Reviewers were instructed to review the user interface of each app, the terms of service, and the app’s privacy policy to check the app against each of the 26 heuristics thoroughly. During the evaluation, if a heuristic did not apply to a particular app, it was scored as “not applicable” and that app was not considered in the analysis of a particular heuristic. (This is a common approach in the use of heuristics in usability evaluation [25].) For example, many apps do not allow data to be shared with SNSs, and it would not make sense to apply H20 to H26 to these apps. The relevance of particular heuristics should, therefore, be made at the discretion of the evaluator, given the particular functionality of the app under consideration.

To assess the interrater reliability in applying the heuristics, 12 apps were chosen at random to be evaluated by a second reviewer. Apps were allocated such that all reviewers had evaluated the work of each of their peers at least once. The interrater reliability was assessed using Cohen kappa [39], which suggested a moderate agreement between raters (kappa=.45). Disagreements between the raters arose primarily from confusion over the apps’ privacy policies, which were often unclear in terms of language and intent. Reviewers discussed these issues to resolve the disagreement and come to a consensus.

The outcome of the analysis was an overall privacy score for each of the 64 apps, with the score calculated by summing the ratings that each app achieved against each of the applicable heuristics, implying that scores should not be interpreted linearly but rather as a reflection of the total number of heuristics that were applicable to the app. (This means that the score can also be expressed as a percentage by calculating the total possible score that an app could have achieved, given the total number of heuristics that are applicable to it.) In the next section, we discuss the results of the heuristic evaluation in terms of differences between app categories and significant effects.

## Results

**Figure 2:**
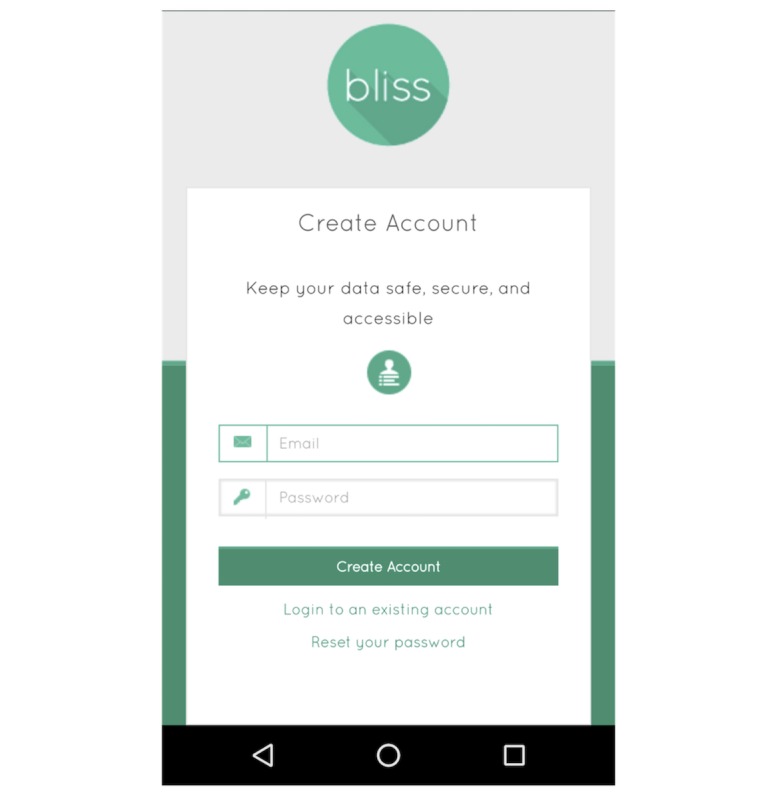
Screenshot of initial login screen of the Bliss app.

Of the 64 examined apps, only 1 failed all of its applicable heuristics. This app, a “gratitude journal” named Bliss (Figure 2), invites users to create an account when first launching the app, but does not offer any information about how user data are handled, which was a common issue among self-tracking apps in general. The gratitude journal Bliss performed poorly in the heuristic evaluation, satisfying none of the heuristics it was tested against. No mHealth apps had a maximum possible score on all applicable heuristics, but one self-tracking app (adhk’s *Timesheet*) performed well on all applicable heuristics. This app only stores data locally on the user’s device, avoiding most of the issues regarding the sharing and storage of data that are covered by the heuristics. The mean heuristic satisfaction (of the maximum possible score) for each app was 46.2% (SD 24.3%), with high variability between apps. To analyze these data, we fit an appropriate cumulative link mixed model (CLMM), an ordinal regression model that exploits the categorical nature of our heuristic scoring, with the ability to account for random effects such as multiple ratings of the same app [40]. This allows for an investigation of the differences between different categories of apps, as well as whether apps for mHealth differ from other types of self-tracking apps in terms of privacy.

**Figure 3:**
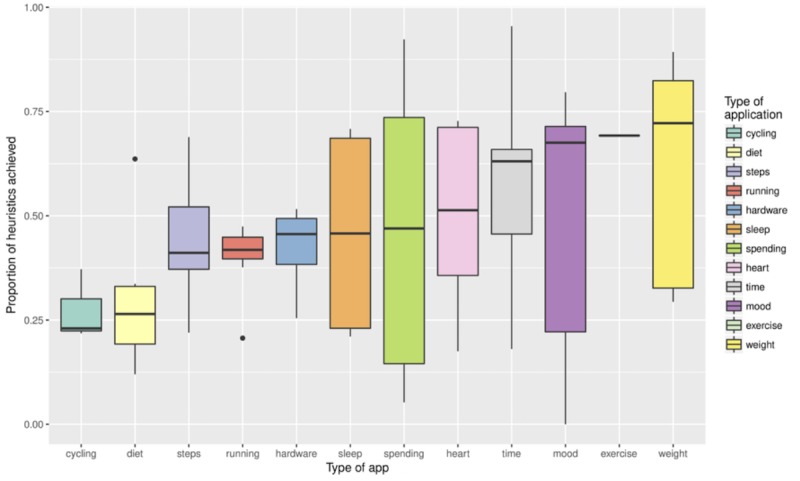
Boxplot showing how performance on the privacy heuristics varies across different types of apps.

While the relationship between the app category (as listed in Table 2) and its performance was variable, the CLMM demonstrated that the type of app was not a useful predictor of its performance. Figure 3 shows the wide variability in performance across apps of different types. Weight-tracking apps performed best, and cycling apps performed worst; however, the type of app is not a significant predictor of performance. One interesting finding was that apps from broadly similar categories (in terms of their purpose and functionality) often scored very differently when evaluated against our heuristics. For example, apps that track “exercise” performed well, achieving a mean of 69.2% (SD 48%) of the maximum heuristic score on average, whereas functionally similar cycling apps were among the worst performers, scoring only a mean of 27.4% (SD 37.9%). This result is surprising given that we might intuitively expect the sensitivity of the data collected by an app to relate to the privacy strategies adopted by its developers.

Following this, we considered whether the maturity of an app’s position in the marketplace led to a better performance. For example, one might imagine that the developers of higher profile and more established apps would have had time to respond to increased scrutiny with better privacy controls. However, our attempts to explore this question using the number of downloads, the average star rating, or the total number of ratings as a proxy for maturity failed to indicate a significant relationship.

**Figure 4:**
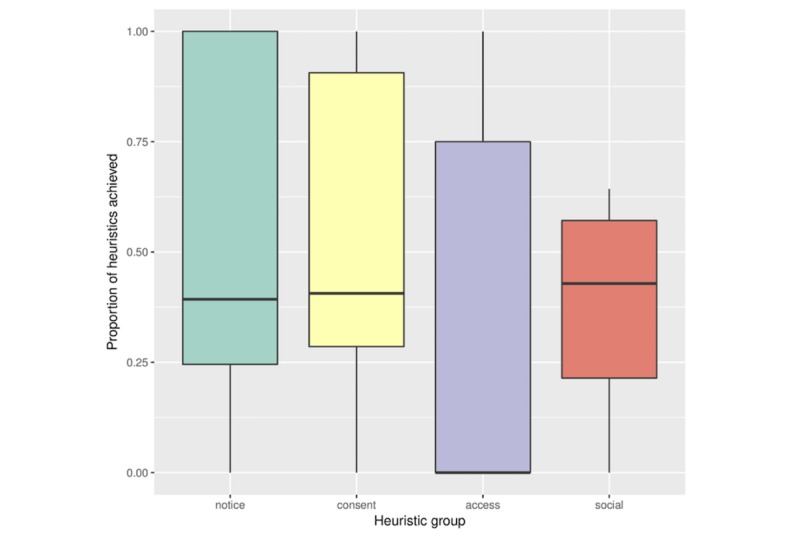
Boxplot showing the differences in performance among the 4 groups of heuristics.

We next explored differences among 4 dimensions of privacy captured by our heuristics, focusing on how the dimensions are upheld at a high level. Figure 4 demonstrates the differences in performance among the 4 categories, which was broadly similar except for access to user data, which was significantly poorer. Most apps failed to offer users sufficient access to their own data, despite this being a fundamental aspect of many data protection regimes. The CLMM showed significantly higher performance for the consent and notice heuristics than for access, but not for social disclosure usability [1.5 (95% CI 0.8 to 2.2), *P*<.001; 1.6 (95% CI 0.9 to 2.3), *P*<.001; and 0.2 (95% CI −0.7 to 1.1), *P*=.63, respectively]. The relatively low scoring of apps on the access dimension can be partly attributed to the dichotomous manner in which apps allow users to access (and, thus, keep a personal record of) their data. We found that apps either allow the user to export all of their data in a range of text formats or offer no export capabilities whatsoever. Some apps restrict the ability to export data unless the user pays money to “unlock” the app or subscribe to a premium tier. We considered a paywall between users and their own data to be unsatisfactory in terms of meeting the heuristics for the analysis.

**Figure 5:**
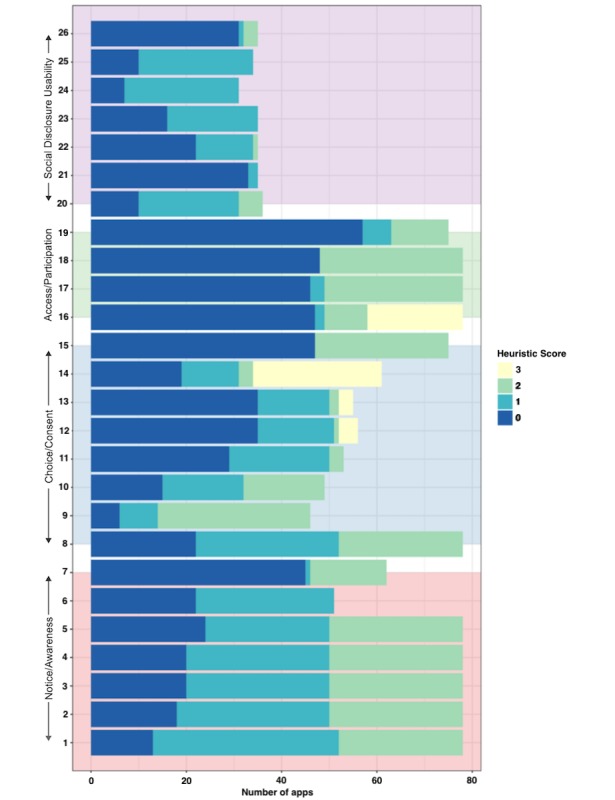
Boxplot showing differences in performance among the 4 groups of heuristics.

Looking closer at the performance of apps with respect to individual heuristics, we observed a great variability in performance. Figure 5 shows the distribution of scores across all heuristics. Note that the horizontal length of bars differs because not all heuristics are applicable to each app. A CLMM suggested significantly poor results for 13 heuristics. The most pertinent examples include the following:

- H19, concerning programmatic access to data, had a particularly low score (−2.5 [95% CI −3.2 to −1.7], *P*<.001). Only 20% (13/64) of apps offered any kind of access to data.
- H21, concerning the ability to share granular QS data with SNSs (−4 [95% CI −5.6 to −2.5], *P*<.001), was permitted by a mere 6% (4/64) of apps.
- H26, concerning the availability of contextual support when making privacy decisions (−3.1 [95% CI −4.3 to −1.9], *P*<.001), was met by 10% (6/64) of apps.

Only 2 heuristics indicated a significant positive effect:

- H9, concerning the requirement for consent processes to be opt-in rather than opt-out (1.4 [95% CI 0.7 to 2.1], *P*<.001), was the best-performing heuristic, with 78% (50/64) of apps scoring above 0.
- H14, concerning the ability to revoke consent for services to use data (1.6 [95% CI 0.8 to 2.3], *P*<.001), was met by 54% (35/74) of apps.

Finally, we compared the performance of mHealth apps with self-tracking apps, which focus on nonhealth data, that is, productivity and time keeping (see Table 2). A CLMM revealed that nonhealth-related self-tracking apps performed significantly better in the heuristic analysis (95% CI 0.1 to 0.7, *P*=.02). This is of concern given that data collected by mHealth apps are likely to be highly personal and individualized, which, combined with the performance of these apps on the heuristic analysis, suggests that the risk of privacy violations may be higher than what is desired.

## Discussion

### Principal Findings

This paper presented a heuristic approach for assessing the privacy features of self-tracking apps. Our heuristics cover 4 key dimensions of information privacy and provide a method for evaluating privacy across a wide range of self-tracking and mHealth apps. We used the heuristics to evaluate 64 popular Android apps that were designed to collect QS data. Our analysis produced several key findings that provide a view on the current state of privacy in self-tracking apps.

First, we found that the majority of apps performed poorly when gauged against our heuristics, with access to user data particularly lacking (see Figure 4). Examples of areas in which the majority of apps scored poorly include providing programmatic access to data (H19), allowing control over the granularity of data when they are shared (H21), and the availability of help and documentation to support decision making (H26). Moreover, we found the category of data collected by the app was not a significant predictor of its privacy performance. This demonstrates that there is no single category of app that performs universally well; rather, the strength of privacy features tends to vary across app categories. In addition, Figure 3 demonstrates that the strength of privacy features often varies *within* app categories. These findings are important because they suggest that privacy stands to be improved across the spectrum of self-tracking apps, warranting the development of standards that can ensure that users’ privacy is upheld in future designs.

The second finding was that app maturity was not a predictor of its strength in terms of privacy. Intuitively, consumers with privacy concerns might favor the services of established market players under the assumption that the apps provided by them are mature and, hence, stronger in terms of privacy [41]. However, our analysis shows that common proxies for an app’s maturity such as the number of downloads or the rating an app has received are not useful predictors of how privacy-preserving an app will be. This again draws attention to the potential for privacy features to be enhanced across the self-tracking app landscape, irrespective of the reputation of an app’s developer. One noteworthy point here is that the measures we used to evaluate an app’s maturity cannot be taken as a ground truth measure, suggesting that this variable should, therefore, be explored more thoroughly in future work.

Our third main finding was that apps classed as tracking data relevant to mHealth (physical activity, mood, and so on) exhibited significantly higher privacy issues than other self-tracking apps. This is of significant concern given that mHealth data have the potential to be highly revealing about a users’ life and, in turn, could lead to harmful or embarrassing situations if the privacy of these data is not upheld. During our evaluation, we noticed that nonmHealth apps tend to store data on users’ devices rather than sharing them with third parties, and it is this practice that gives them a higher privacy score. It is not clear from this analysis, however, why this disparity has emerged in the design of these apps. A cause of concern is that health data may be perceived to be of significant commercial value by designers, so a requirement to share data with vendors is built into the design of such apps to exploit this. In turn, designers might gloss over this issue by “marketing” the sharing of data as something that is solely beneficial to users, for example, to allow synchronization of data between devices, without drawing attention to the associated privacy issues. The inadequate consent mechanisms frequently employed in self-tracking apps mean that users cannot make an informed decision about whether this trade-off is acceptable.

Overall, the importance placed on privacy in QS and mHealth apps is highly variable, and it may, therefore, be difficult for end users to make informed decisions about which apps will provide the functionality they desire while meeting their privacy requirements. We believe that drawing attention to these issues should motivate the development of standards and guidelines that can ensure that future self-tracking apps are stronger with respect to upholding users’ privacy. In addition, our study provides evidence on the value of our heuristic evaluation approach for assessing privacy issues more generally. Using the heuristics will allow designers to consider key privacy concerns when developing and evaluating self-tracking apps in the future. The heuristics should also orient the designers to look toward the regulatory landscape for guidance on privacy-upholding features. Furthermore, the heuristics may guide data controllers in conducting impact assessments for privacy and data protection, such as those mandated by Article 35 of the EU’s GDPR [20], which state that the review must be systematic but offer no guidance as to which aspects to prioritize or how to ensure coverage. The heuristics could be used to perform an initial “triage” assessment of data protection, with low scores in sections 1 and 2 indicating areas of concern. This could then flag that a more detailed impact assessment is necessary.

While we have applied the heuristics to a set of 64 self-tracking apps, we note that the heuristics were created on the basis of the wider regulatory landscape, and thus, they might also be generalizable to other types of apps that collect data from users. However, our study was focused on self-tracking apps, and thus, we see this as an area that should be explored in future work.

### Reviewing the Heuristics

Our heuristics were designed to address 4 key questions regarding user privacy. We now consider the extent to which the apps we examined satisfy these 4 questions.

#### Do Users Know What Will Happen to Their Data Before Using an App?

The 7 *notice* heuristics (H1 to H7) address fundamental descriptive aspects of a user’s relationship with a particular service in terms of who is responsible for the user’s data, what the service will do with it, and who the data will be shared with. Of the 80% (51/64) apps in our sample that collected personal data from users, only 29 included terms of service or a privacy policy to explain how data will be used and none required these policies to be read or comprehended before proceeding with registration. Basic information was missing in many cases, such as the name of the organization collecting the data (25% of apps, 16/64) and who such data might be shared with (39% of apps, 25/64). Many apps allow registration via several routes, using the single sign-on app programming interfaces provided by companies such as Facebook or Google to simplify the registration process for users and to provide the developer with a valuable connection to a user’s Web-based identity. However, many apps that we examined exhibited a pattern whereby using such a registration route bypassed the usual exposure to an app’s terms of service or consent to marketing communications, despite the potential added sensitivity of giving the providers access to their social network identity. High-profile apps from Nike+, Endomondo, and MapMyRide all exhibited this behavior. While Fitbit includes a link to its terms of service and privacy policy from within the app, these are dated 2011 and 2012, respectively, despite both documents having been updated in 2015 and 2016.

While it is well understood that lengthy terms of service are often not sufficiently readable [42] and that most people do not read them [43], it is of concern that so many apps, including those from QS market leaders, fail to provide information about the way their services function *in situ*. With data protection regimes such as the EU’s GDPR strengthening the requirements for clear privacy notices at the time of data collection, it is evident that this is being regularly subverted by many self-tracking apps. US-based companies can self-certify their compliance with the Privacy Shield framework to signify that data exchanged between the United States and EU broadly correspond with EU data protection requirements. Notably, Under Armour, which provides MyFitnessPal, Endomondo, and MapMyRide, among a number of other successful self-tracking apps and devices, has a Privacy Shield certification that explicitly excludes these apps [44].

#### Do Users Have Control Over Their Data?

**Figure 6:**
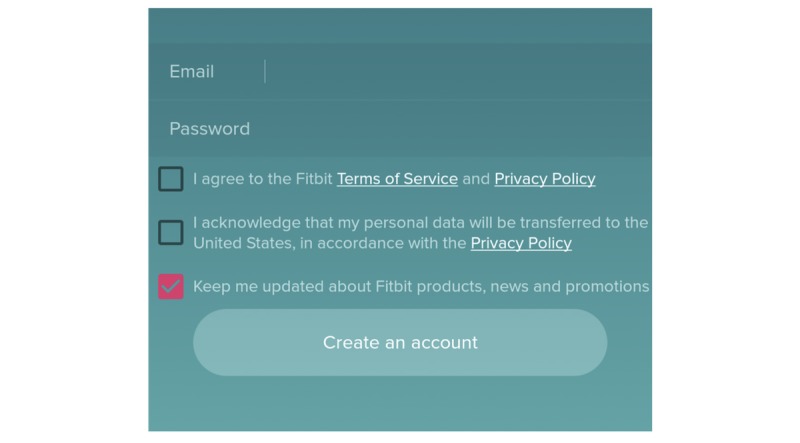
Partial screenshot of the Fitbit Android app account creation page.

The 8 *choice and consent* heuristics (H8 to H15) concern the ability of users to sanction particular uses of their data and revoke consent from the app or connected services and devices. Most apps require an explicit form of consent before collecting data; however, the extent to which such consent is meaningful or sufficient is difficult to determine, with people’s privacy attitudes frequently changing and potentially voiding their previous consent decisions [45], with a sustained approach to acquiring the consent necessary to renegotiate this relationship over time [46]. In some instances, the consent process includes a combination of opt-in, opt-out, or prefilled elements, which can be confusing for users and lead to oversharing of information or exposure to unexpected marketing communications, as shown in Fitbit’s sign-up interface in Figure 6. In addition, most apps reserve the ability to renegotiate the user’s relationship with the service unilaterally by changing terms of service or privacy policies without gaining explicit consent from users. Even if a user is satisfied with the way a company operates its service at the time of registration, the service can change significantly without the user’s knowledge, thereby compromising the value of his or her consent. While some apps, such as Nike+, commit to notifying users and gaining consent if the use of personal data changes, this is still uncommon among self-tracking apps. While 36% of apps examined only keep data on the user’s device, among those that involve sharing data with a remote provider, 63% do not provide users with control over where their data are stored, requiring users to trust that the vendor, or their choice of storage provider, can be trusted upon. Such efforts to ensure that data provided by users remain proprietary and siloed is a concern, which is further confirmed in our study of the third dimension of privacy.

#### Do Users Have Access to the Data They Have Provided?

The 4 *access and participation* heuristics (H16 to H19) concern users’ ability to extract the data they have provided from an app. Data portability is an increasingly sensitive issue in Internet services, with data protection regimes increasingly acknowledging portability as a right. Despite the fact that self-tracking requires the disclosure of potentially sensitive data to a range of data controllers, most services do not give people unfettered access to their own data, as indicated by the low scores for these access heuristics reported in Figure 4. Only 26% of services allow people to export all of their data, and 60% provide no means of exporting data. Our finding that only 16% apps provide programmatic access to data through public app programming interfaces supports the conclusion that mHealth and QS services are encouraging people to silo their data to prevent portability.

#### Can Users Control Their Disclosures to Third Parties?

While many services are keen to function as self-contained entities, some permit users to disclose their activity to third-party services such as SNSs. Such functionality has benefits for users who use SNSs to build social capital [47], which can be reinforced through the presentation of self-tracking activity such as weight loss or running performance; this, in turn, can support behavioral change [48]. For the developers of self-tracking services, this is a simple way to promote their service through evangelical users and associate their brand with positive behaviors. Over 40% of apps allow users to publish their activity to third-party services and meet most of the fundamental usability measures. Only 11% of apps, however, provide contextual privacy help, such as explaining the effect of sharing information with different audiences. Most of the heuristics in this category (H20 to H26) had high scores for the apps examined, which, in part, may be attributed to the use of the authentication software development kit provided by major SNSs, such as Facebook and Twitter, which provide their own native privacy controls and audience selectors in response to ongoing privacy issues with their platforms.

As self-tracking apps continue to evolve, they will incorporate new types of sensors, a greater fidelity of captured data, the ability to provide richer analysis, and more accurate inferences. Current apps are mostly delivered through smartphones and wristwear; however, this is often associated with usability issues due to small or absent displays. Therefore, we can anticipate that in the near future, sensors will increasingly be embedded in biological and interfaceless apps, reducing the usability barriers to adopting such technologies, while potentially introducing new privacy risks when it becomes harder to configure the appropriate sharing of information. We, therefore, propose that these heuristics can be used as a form of certification attached to the marketing of products in app stores and other channels. This would allow people to compare the privacy characteristics of apps that offer similar functionality and encourage developers to incorporate innovative privacy-preserving functionality, thus, treating privacy as a value-adding marketable feature.

### Limitations

This study has several limitations. First, using the results of a survey from a QS meetup group was sufficient to capture the types of data people are interested in tracking. It is not clear, however, whether the self-selected QS community is representative of the cohort of users who use such technologies but may not consider themselves self-trackers, nor have any affinity with self-tracking as a practice. For example, by far, the most popular app that we examined was Simple Design Ltd’s *Period Calendar*, which was 38% more popular than the next-ranked app *MyFitnessPal*. The former was only included in our scrape of Google Play as it matched the “mood” keyword. Considering the popularity of the app, it is plausible that the QS community is significantly biased toward men, and therefore, our analysis may have omitted some self-tracking apps. Similarly, regional differences may have caused us to miss some apps. Our study used the UK Google Play Store, and the list of apps returned may differ from that produced if the scrape was performed in another region. For example, the financial tracking app *Mint* [49] is only available in the United States, and so it did not appear in our list of apps, despite having nearly 10 million downloads.

In terms of our findings, we have designed the heuristics and the process of obtaining and reviewing apps to be reproducible, but we do not yet know how robust the heuristics are to changes in technology, as the privacy implications and usability challenges we observe may be tightly coupled to the modalities of smartphones and wearables, which currently dominate the self-tracking landscape. Cohen kappa for our interrater reliability only showed moderate agreement, which may be attributed to the varying interpretation of legal language in privacy policies and term & conditions as well as the fact that only on a subset of apps was compared by pairs of raters. Increasing the number of raters per app might address this issue.

In addition, the finding that mHealth apps performed worse on privacy is worthy of deeper investigation given the aforementioned possibility of selection bias in our app sample and differences in sample size between the mHealth and nonmHealth app categories. In addition, it would be helpful to consider how the heuristics can be reconciled with the fact that different aspects of privacy can become more or less relevant to users depending on their context [50,51]. The heuristics in our study were applied by expert evaluators without attending to the context, primarily because the heuristics are designed to embody a set of issues that should be applicable across a range of settings (eg, the usability of disclosure controls). Nevertheless, understanding whether users might actually desire less stringent privacy controls in certain contexts is an area for future work.

In terms of our method, the heuristics can be time consuming to apply because they require a close reading of terms of service. In addition, they require many functional routes of an app to be explored in order to identify discrepancies. In future work, we plan to investigate whether natural language processing can be used to parse and semiautomatically apply some of the heuristics to the terms of service and privacy policies. Work by Slavin et al [52] has focused on detecting privacy policy violations in Android app code, and similar techniques could be used to automatically apply the privacy policy heuristics as well as those concerned with exporting data.

### Conclusions

In this paper, we have introduced a novel heuristic evaluation method for examining the state of privacy in QS apps. We found that the majority of apps do not meet our privacy criteria, including notification of fundamental data protection characteristics, or the criteria on ability to export user data. High-profile apps are among those that exhibit poor privacy behaviors, which can make it difficult for users to make informed choices about which apps to trust with their data. Our heuristics can provide designers with a resource to maintain privacy in the design of self-tracking services and avoid common pitfalls, which can engender mistrust or lead to privacy issues. As the heuristics were guided by both the EU and US regulatory environment, they may also help guide data controllers to perform impact assessments for both privacy and data protection. We have provided the tools and documentation necessary to replicate our findings and confirm the usability of the heuristics and allow the evolving privacy landscape to be evaluated. In future work, we will examine the *usefulness* of the heuristics by using them to capture people’s privacy preferences and recommend services that meet their requirements.

## Appendix

Multimedia Appendix 1 Quantified Self Privacy Heuristics v1.

Multimedia Appendix 2 London Quantified Self Tool Use Survey 2014.

Multimedia Appendix 3 Apps chosen for heuristics evaluation.

## Abbreviations

CLMM: cumulative link mixed model
EU: European Union
GDPR: General Data Protection Regulation
mHealth: mobile health
QS: quantified self
SNS: social networking site
