# Supplementary material for: Assessing the Privacy of mHealth Apps for Self-Tracking: Heuristic Evaluation Approach
Source: JMIR Mhealth Uhealth. 2018 Oct 22;6(10):e185. doi: 10.2196/mhealth.9217 (PMC6231850; doi:10.2196/mhealth.9217)
Supplement: Multimedia Appendix 1 [file mhealth_v6i10e185_app1.pdf]

Quantified self privacy heuristics.

Version 1

October 23, 2017

1 Notice/awareness

H1 Before data are shared with a remote actor, the entity collecting the data is explicitly identified [0-2]

- 0: Entity is not identified
- 1: Entity is identified
- 2: No remote sharing

H2 Before data are shared with a remote actor, the uses of the data are explicitly identified [0-2]

- 0: Uses are not identified
- 1: Uses are identified
- 2: No remote sharing

H3 Before data are shared with a remote actor, the potential recipients are explicitly identified [0-2]

- 0: Recipients are not identified
- 1: Recipients are identified
- 2: No remote sharing

H4 The nature of the data collected and by which means are explicitly identified [0-2]

- 0: Nature of data collection not identified
- 1: Nature of data collection is identified
- 2: No remote sharing

H5 Steps taken to ensure confidentiality, integrity, and quality of data are explained [0-2]

- 0: Steps taken not identified
- 1: Steps taken identified
- 2: No remote sharing

H6 For those of above satisfied, notice is sufficiently explicit [0-4]

- 0: No data usage explained
- 1: No requirement for terms of service/privacy policies to be read: eg. links which opens in browser

- 2: Requirement for policies to be read: eg. shown in situ before consent acquired and must be scrolled through
- 3: Must be read and data usage policy described in non-legal language
- 4: Must be read and comprehension of data usage is assessed

H7 Can control when data are used for non-operational secondary use, such as marketing or research. [0-2]

- 0: No control over who receives which data
- 1: Must explicitly allow data to be shared for secondary uses
- 2: Can control which data is shared with other actors, or no data are shared.

2 Choice/consent

H8 Consent acquired before data shared with remote actor [0-2]

- 0: Consent not acquired
- 1: Consent acquired
- 2: No remote sharing

H9 Consent is explicitly opt-in: no pre-ticked checkboxes etc. [0-2]

- 0: Consent flow mixes opt-in and opt-out elements: eg. “check to agree” alongside “check to opt-out of data sharing”
- 1: Consent includes pre-filled elements, for agreement or sharing with third parties
- 2: Consent is opt-in: requires checkbox ticked or “agree” button clicked, etc.

H10 Can choose which data types are automatically collected from sensors or other sources eg. connect a finance app to a single bank account, or track steps but not heart rate [0-2]

- 0: No controls over collection of specific data types
- 1: Must consent to individual sensors being engaged or data types collected
- 2: Can revoke ability to track particular data types at any time

H11 Data collection consent is dynamic: if new types of data are being collected, consent is renewed in situ. [0-2]

- 0: No dynamic consent acquisition
- 1: Consent acquired before a new sensor is engaged or data type collected, eg. using device permission APIs
- 2: Contextual permission includes a meaningful justification for why new sensor is required

H12 Data processing consent is dynamic: if the purpose of processing changes, consent is renewed. [0-3]

- 0: No dynamic consent
- 1: Notification of processing changes (implied consent)
- 2: Consent acquired after processing changes
- 3: Purpose of change explained clearly and consent acquired

H13 Data distribution consent is dynamic: if the actors data are distributed to changes, consent is renewed. [0-3]

- 0: No dynamic consent
- 1: Notification of distribution changes (implied consent)
- 2: Consent acquired after distribution changes
- 3: Purpose of change explained clearly and consent acquired

H14 Consent to store and process data can be revoked at any time: with the service, and any other actors [0-3]

- 0: No consent revocation
- 1: Consent can be revoked by contacting support
- 2: Consent with third parties can only be revoked from those third parties
- 3: Consent with service and all third party actors can be revoked from within the app/service

H15 Can control where data are stored [0-2]

- 0: Data are stored on vendor servers or choice of cloud provider, with no user control over this
- 1: Can choose which provider stores data (eg. Amazon vs Google)
- 2: Can choose to only store data on-device and selectively disclose to other actors

3 Access/participation

H16 All raw collected data can be extracted from the service (in-app or via vendor's website) [0-3]

- 0: No method of extracting data
- 1: Data can be extracted in aggregate form (eg. daily totals/averages)
- 2: Subset of *raw* data can be extracted (eg. only certain data types, or limited time range)
- 3: All raw data can be extracted

H17 All data are available in standard text formats (CSV, XML, JSON, GPX, etc) [0-2]

- 0: Data are not available
- 1: Data available in proprietary or binary formats
- 2: Data available in standard text formats

H18 Data extraction is available from within the service: eg. without raising a request with support [0-2]

- 0: No method for extracting data
- 1: Data can be extracted by contacting the service provider
- 2: Data can be extracted at any time from within the service (even if there is a delay while an archive is generated)

H19 Programmatic access to data is possible: eg. APIs are exposed [0-2]

- 0: No programmatic access
- 1: Limited programmatic access (aggressively rate limited, subset of data exposed)
- 2: Full programmatic access (eg. H16 and H17 can be satisfied programmatically)

4 Social disclosure usability

H20 Privacy controls are per-disclosure: eg. individual workouts can be published to a social network site, not relying solely on global defaults [0-2]

- 0: No per-disclosure controls
- 1: Can choose to disclose individual actions
- 2: Can choose the audience for individual disclosures

H21 Privacy controls allow granular sharing of data types: for example, when sharing a workout, the distance can be shared but not the pace. [0/1]

- 0: No granular controls
- 1: Can choose which attributes of an event are shared

H22 Error prevention: is explicit confirmation acquired before a disclosure? [0-2]

- 0: No confirmation acquired
- 1: Confirmation needed before disclosure
- 2: Confirmation needed and able to quickly revoke an erroneous disclosure (this is distinct from being able to revoke/correct historic data)

H23 Minimise user memory load: Effects of a disclosure are visible throughout the disclosure flow (ie. memory of earlier decisions not required) [0/1]

- 0: Content or audience of a disclosure is occluded at points during disclosure process
- 1: Content and audience of a disclosure is available during the disclosure process

H24 Minimalist: During the disclosure flow no extraneous information (such as adverts or irrelevant UI elements) is displayed [0/1]

- 0: Extraneous information displayed during disclosure process
- 1: All information presented during disclosure is relevant to configuring the disclosure

H25 Consistency: Information shown during the disclosure flow is consistent with the effect of the disclosure [0/1]

- 0: The effect of the disclosure does not match what was presented during the disclosure flow, whether over or under-sharing.
- 1: The effect of the disclosure is consistent with the information presented during the disclosure process, with no more, or less, data being shared than the disclosure flow suggests.

H26 Help and documentation: Contextual help with making privacy decisions is available [0-2]

- 0: No contextual support in disclosure or privacy settings UIs
- 1: Linked to external support (eg. documentation which launches in browser)
- 2: Documentation is natively embedded within the app
