# Supplementary material for: Assessing the Privacy of mHealth Apps for Self-Tracking: Heuristic Evaluation Approach
Source: JMIR Mhealth Uhealth. 2018 Oct 22;6(10):e185. doi: 10.2196/mhealth.9217 (PMC6231850; doi:10.2196/mhealth.9217)
Supplement: Multimedia Appendix 3 [file mhealth_v6i10e185_app3.pdf]

## Appendix 2: Apps chosen for heuristic evaluation

| Package name | Name | Version tested | Keywords | Group | Min Downloads | Max Downloads |
|---|---|---|---|---|---|---|
| com.popularapp.periodcalendar | Period Tracker, My Calendar | 1.5907.145 | [mood] | mood | 50000000 | 100000000 |
| com.myfitnesspal.android | Calorie Counter - MyFitnessPal | 6.6 | [weight, diet] | diet | 50000000 | 100000000 |
| com.runtastic.android | Runtastic Running & Fitness | 7.1.1 | [running, running, exercise, workouts] | running | 10000000 | 50000000 |
| com.nike.plusgps | Nike+ Run Club | 2.3.2 | [running, running] | running | 10000000 | 50000000 |
| com.fitnesskeeper.runkeeper.pro | RunKeeper - GPS Track Run Walk | 7.3.2 | [walking, running, running] | running | 10000000 | 50000000 |
| com.endomondo.android | Endomondo - Running & Walking | 12.12.0 | [walking, running, exercise, workouts, cycling] | running | 10000000 | 50000000 |
| si.modula.android.instantheartrate | Instant Heart Rate Monitor | 5.36.2835 | [heartrate, heart rate] | heart | 10000000 | 50000000 |
| cc.pacer.androidapp | Pedometer & Weight Loss Coach | p2.16.1 | [weight, steps, walking] | weight | 10000000 | 50000000 |
| com.urbandroid.sleep | Sleep as Android | 20161122 | [sleep, sleep] | sleep | 10000000 | 50000000 |
| com.strava | Strava Running and Cycling GPS | 5.10.0 | [running, running, cycling] | running | 5000000 | 10000000 |
| com.tayu.tau.pedometer | Pedometer | 5.19.0 | [steps, walking] | steps | 10000000 | 50000000 |
| com.fitbit.FitbitMobile | Fitbit | 2.4.3 | [steps, exercise, workouts] | hardware | 10000000 | 50000000 |
| com.sec.android.app.shealth | S Health | 5.4.1.0003 | [steps, heart rate] | steps | 100000000 | 500000000 |
| com.kpmoney.android | AndroMoney ( Expense Track ) | 2.9.0 | [spending, income] | spending | 1000000 | 5000000 |
| com.google.android.apps.fitness | Google Fit - Fitness Tracking | 1.62.06-108 | [steps, walking, exercise, workouts] | steps | 10000000 | 50000000 |
| com.stt.android | Sports Tracker Running Cycling | 3.9.17 | [exercise, workouts, cycling] | running | 5000000 | 10000000 |
| com.sonymobile.lifelog | Lifelog | 3.0.A.4.34 | [steps] | steps | 10000000 | 50000000 |
| com.runtastic.android.heartrate.lite | Runtastic Heart Rate Monitor | 2.3 | [heartrate, heart rate] | heart | 5000000 | 10000000 |
| com.mapmyrun.android2 | Run with Map My Run | 16.12.1 | [running, running] | running | 5000000 | 10000000 |
| com.noom.walk | Noom Walk Pedometer | 1.4.0 | [steps, walking] | steps | 10000000 | 50000000 |
| com.dietcoacher.sos | My Diet Coach - Weight Loss | 4.4.1 | [weight, diet] | weight | 10000000 | 50000000 |
| tools.bmirechner | BMI Calculator & Weight Loss | 3.7.9 | [weight] | weight | 10000000 | 50000000 |
| com.todoist | Todoist: To-Do List, Task List | 11.2.4 | [time keeping] | time | 10000000 | 50000000 |
| com.weightwatchers.mobile | Weight Watchers Mobile | 5.3.1 | [weight, eating] | weight | 5000000 | 10000000 |
| com.mapmywalk.android2 | Walk with Map My Walk | 16.11.1 | [walking] | steps | 1000000 | 5000000 |
| com.runtastic.android.roadbike.lite | Runtastic Road Bike GPS App | 3.0.2 | [cycling] | cycling | 1000000 | 5000000 |

| | | | | | | |
|---|---|---|---|---|---|---|
| com.bookmark.money | Money Lover - Money Manager | android-3.5.86 | [spending] | spending | 1000000 | 5000000 |
| com.lib.cwmoney | CWMoney 2.21 Expense Track | 2.23 | [spending] | spending | 1000000 | 5000000 |
| com.runtastic.android.sleepbetter.lite | Sleep Better with Runtastic | 2.3 | [sleep, sleep] | sleep | 1000000 | 5000000 |
| com.runtastic.android.pedometer.lite | Runtastic Pedometer Step Count | 1.6.2 | [steps] | steps | 5000000 | 10000000 |
| monitoryourweight.bustan.net | Monitor Your Weight | 4.8.8 | [weight] | weight | 5000000 | 10000000 |
| com.mapmyride.android2 | Map My Ride GPS Cycling Riding | 12.12.1 | [cycling] | cycling | 1000000 | 5000000 |
| com.sillens.shapeupclub | Lifesum - The Health Movement | 4.2.0 | [diet] | diet | 5000000 | 10000000 |
| com.monefy.app.lite | Monefy - Money Manager | 1.7.8 | [spending] | spending | 1000000 | 5000000 |
| com.realbyteapps.moneymanagerfree | Money Manager Expense & Budge | 3.6.12 FO | [spending] | spending | 1000000 | 5000000 |
| com.fitnow.loseit | Lose it! | 7.2.5 | [eating, diet] | diet | 5000000 | 10000000 |
| com.expensemanager | Expense Manager | 3.2.3 | [spending] | spending | 1000000 | 5000000 |
| com.droid4you.application.wallet | Wallet - Budget Tracker | 4.3.3 | [spending] | spending | 1000000 | 5000000 |
| com.gmail.jmartindev.timetune | TimeTune - Conquer Your Time | 2.0.3 | [time keeping] | time | 1000000 | 5000000 |
| com.northcube.sleepcycle | Sleep Cycle alarm clock | 1.4.1192 | [sleep, sleep] | sleep | 500000 | 1000000 |
| com.yazio.android | Calorie Counter & Diet Tracker | 3.1.0.3 | [diet] | diet | 1000000 | 5000000 |
| com.azumio.android.sleeptime | Sleep Time Smart Alarm Clock | 1.36.1042 | [sleep] | sleep | 1000000 | 5000000 |
| de.aktiwir.aktibmi | Weight Log & BMI Calculator | 1.44 | [weight] | weight | 1000000 | 5000000 |
| net.daylio | Diary - Mood Tracker | 1.8.3 | [mood] | mood | 100000 | 500000 |
| com.sparkpeople.androidtracker | Calorie Counter & Diet Tracker | 4.55 | [diet] | diet | 1000000 | 5000000 |
| com.intuit.quickbooks | QuickBooks Accounting+Invoice | 5.1.2 | [timekeeping] | time | 1000000 | 5000000 |
| jp.co.recruit.mtl.beslim | Weight Loss Tracker - RecStyle | 3.1.7 | [body fat] | weight | 1000000 | 5000000 |
| com.aadhk.time | Timesheet | 1.6.0.9 | [time keeping] | time | 500000 | 1000000 |
| fi.polar.polarflow | Polar Flow - Activity & Sports | 3.3.1 | [exercise, workouts] | hardware | 1000000 | 5000000 |
| arproductions.andrew.worklog | Work Log | 2.4.2 | [timekeeping] | time | 500000 | 1000000 |
| com.heartrate.monitor | Heart Rate Monitor | 3.684 | [heartrate, heart rate] | heart | 1000000 | 5000000 |
| com.supersimpleapps.heart_rate_monitor_new | Unique Heart Rate Monitor | 1.26 | [heartrate, heart rate] | heart | 1000000 | 5000000 |
| com.dungelin.heartrate | Heart Rate Monitor & Tracker | 2.4.4 | [heartrate, heart rate] | heart | 500000 | 1000000 |
| com.rauscha.apps.timesheet | Timesheet - Time Tracker | 2.1.3 | [time keeping] | time | 500000 | 1000000 |
| comm.cchong.BloodAssistant | iCare Health Monitor (BP & HR) | 3.3.3.170207 | [heartrate, heart rate] | heart | 500000 | 1000000 |
| com.soh | Secret Of Happiness | 2.1.0 | [happiness] | mood | 100000 | 500000 |

| | | | | | | | |
|---|---|---|---|---|---|---|---|
| com.livestrong.tracker | MyPlate Calorie Tracker | 2.9.0 | [eating] | diet | 100000 | 500000 |
| com.bliss.phonegap | Gratitude Journal | 2.0.6 | [happiness] | mood | 100000 | 500000 |
| com.moodtools.moodtools | MoodTools - Depression Aid | 2.2.8 | [mood] | mood | 100000 | 500000 |
| com.moodtrak.diary | Moodtrack Diary: Mood Tracker | 3.2 | [mood] | mood | 50000 | 100000 |
| de.aktiwir.aktifit | Simple Workout Log - aktiFit | 2.6 | [exercise, workouts] | exercise | 10000 | 50000 |
| com.xiaomi.hm.health | Mi Fit | 2.2.9 | [hardware] | hardware | | |
| com.jawbone.upopen | UP - Smart Coach for Health | 4.27.0 | [hardware] | hardware | | |
